# Supplementary material for: Information sharing and channel structure in e-commerce supply chain considering data-driven marketing
Source: PLoS One. 2025 Sep 8;20(9):e0328040. doi: 10.1371/journal.pone.0328040 (PMC12416664; doi:10.1371/journal.pone.0328040)
Supplement: S1 File — (PDF) [file pone.0328040.s001.pdf]

Appendix A

A.1. Equilibrium solutions in the AN case are

$$w^{\mathrm{AN}*}=\frac{k(1-\lambda)(k(6k-2(2+k)\lambda+\lambda^2)-2(1-\lambda))a_0}{2(k(3-\lambda)-1)(1+k-\lambda)(2k-\lambda)}$$

$$q_R^{\mathrm{AN}*}=\frac{k(2-\lambda)(1-\lambda)a_0}{2(k(3-\lambda)-1)(1+k-\lambda)}+\frac{(2k-\lambda)\xi\sqrt{1-\sigma^2}\sigma_a}{k(2-\lambda)}$$

$$q_M^{\mathrm{AN}*}=\frac{k(1+k(3-\lambda)-\lambda)a_0}{2(k(3-\lambda)-1)(1+k-\lambda)}$$

$$e^{\mathrm{AN}*}=\frac{(2-(2-k(3-\lambda))\lambda)a_0}{2(k(3-\lambda)-1)(1+k-\lambda)}+\frac{\xi\sqrt{1-\sigma^2}\sigma_a}{k}$$

$$\Pi_M^{\mathrm{AN}*}=\frac{k^2(1-\lambda)(4+6k-5\lambda-2k\lambda+\lambda^2)a_0^2}{4(k(3-\lambda)-1)(1+k-\lambda)(2k-\lambda)}$$

$$\Pi_P^{\mathrm{AN}*}=\frac{A}{8(1+k(3-\lambda))^2(1+k-\lambda)^2(2k-\lambda)}+\frac{(k(2+\lambda)^2-4\lambda-4k^2\lambda)(1-\sigma^2)\sigma_a^2}{2k^2(2-\lambda)^2}$$

where, $A=k(4k^4(3-\lambda)^2\lambda+4(1-\lambda)^2\lambda+8k^3(3-\lambda)\lambda(2-(4-\lambda)\lambda)-8k(1-\lambda)\lambda(2-(4-\lambda)\lambda)+k^2(8-(3-\lambda)\lambda(12+\lambda(2-3(5-\lambda)\lambda))))a_0^2$.

A.2. Equilibrium solutions in the AS case are

$$w^{\mathrm{AS}*}=\frac{k(1-\lambda)(k(6k-2(2+k)\lambda+\lambda^2)-2(1-\lambda))(a_0+\xi\sqrt{1-\sigma^2}\sigma_a)}{2(k(3-\lambda)-1)(1+k-\lambda)(2k-\lambda)}$$

$$q_R^{\mathrm{AS}*}=\frac{k(2-\lambda)(1-\lambda)(a_0+\xi\sqrt{1-\sigma^2}\sigma_a)}{2(k(3-\lambda)-1)(1+k-\lambda)}$$

$$q_M^{\mathrm{AS}*}=\frac{k(1+k(3-\lambda)-\lambda)(a_0+\xi\sqrt{1-\sigma^2}\sigma_a)}{2(k(3-\lambda)-1)(1+k-\lambda)}$$

$$e^{\mathrm{AS}*}=\frac{(2+(k(3-\lambda)-2)\lambda)(a_0+\xi\sqrt{1-\sigma^2}\sigma_a)}{2(k(3-\lambda)-1)(1+k-\lambda)}$$

$$\Pi_M^{\mathrm{AS}*}=\frac{k^2(1-\lambda)(4+6k-5\lambda-2k\lambda+\lambda^2)(a_0^2+(1-\sigma^2)\sigma_a^2)}{4(k(3-\lambda)-1)(1+k-\lambda)(2k-\lambda)}$$

$$\Pi_P^{\mathrm{AS}*}=\frac{B}{8(1+k(3-\lambda))^2(1+k-\lambda)^2(2k-\lambda)}$$

where, $B=k(4k^4(3-\lambda)^2\lambda+4(1-\lambda)^2\lambda+8k^3(3-\lambda)\lambda(2-(4-\lambda)\lambda)-8k(1-\lambda)\lambda(2-(4-\lambda)\lambda)+k^2(8-(3-\lambda)\lambda(12+\lambda(2-3(5-\lambda)\lambda))))(a_0^2+(1-\sigma^2)\sigma_a^2)$.

Appendix B

B.1. Proof of Proposition 1

(a)We compare $e^{\mathrm{NS}*}$ and $e^{\mathrm{NN}*}$, and we obtain the following: $e^{\mathrm{NS}*}-e^{\mathrm{NN}*}=-\frac{\xi\sqrt{1-\sigma^2}\sigma_a}{2(2k-1)}<0$.

(b)We compare $\mathrm{w}^{\mathrm{NS}*}$ and $w^{\mathrm{NN}*}$, and we obtain the following: $\mathrm{w}^{\mathrm{NS}*}-w^{\mathrm{NN}*}=\frac{1}{2}\xi\sqrt{1-\sigma^2}\sigma_a>0$.

(c)We compare $q_R^{\mathrm{NS}*}$ and $q_R^{\mathrm{NN}*}$, and we obtain the following: $q_R^{\mathrm{NS}*}-q_R^{\mathrm{NN}*}=-\frac{k\xi\sqrt{1-\sigma^2}\sigma_a}{2(2k-1)}<0$.

(d)We compare $\Pi_M^{\mathrm{NS}*}$ and $\Pi_M^{\mathrm{NN}*}$, and we obtain the following: $\Pi_M^{\mathrm{NS}*}-\Pi_M^{\mathrm{NN}*}=\frac{k(1-\sigma^2)\sigma_a^2}{4(2k-1)}>0$. We compare $\Pi_P^{\mathrm{NS}*}$ and $\Pi_P^{\mathrm{NN}*}$, and we obtain the following: $\Pi_P^{\mathrm{NS}*}-\Pi_P^{\mathrm{NN}*}=-\frac{3k(1-\sigma^2)\sigma_a^2}{8(2k-1)}<0$. This completes the proof. □

B.2. Proof of Proposition 2

(a)We compare $e^{\mathrm{ES}*}$ and $e^{\mathrm{EN}*}$, and we can show that $e^{\mathrm{ES}*}-e^{\mathrm{EN}*}$ is equal to a positive factor multiplied by $g_1=-(k(3k-4)+\eta+5k\eta-2\eta^2)$. Given $k_{min}=\max\{\frac{1}{2},\frac{1}{3}(2-3\eta+2\sqrt{1-3(1-\eta)\eta}),1-\eta,\frac{4-5\eta}{3}\}$, we obtain $g_1<0$.

(b)We compare $w^{\mathrm{ES}*}$ and $w^{\mathrm{EN}*}$, and we can show that $w^{\mathrm{ES}*}-w^{\mathrm{EN}*}$ is equal to a positive factor

multiplied by $g_2 = 3k^2 - 4k(1-\eta) + (2-3\eta)\eta$. Given k, we can look at g_2 as a quadratic function of k and g_2 is convex. We let $g_2 = 0$, and we obtain the two solutions as $\frac{1}{3}(2 - 2\eta - \sqrt{4 - \eta(14 - 13\eta)})$ and $\frac{1}{3}(2 - 2\eta + \sqrt{4 - \eta(14 - 13\eta)})$. There are two cases.

Case1: When 0< $\eta < \frac{5}{6}$, the two solutions are all smaller than k_{min}, thus we require $g_2 > 0$.

Case2: When $\frac{5}{6} < \eta < 1$, the second solution is greater than k_{min}, thus there are two subcases: (i)When $k_{min} < k < k_1$, we require $g_2 < 0$.(ii) When $k > k_1$, we require $g_2 > 0$.Where $k_1 = \frac{1}{3}(2 - 2\eta + \sqrt{4 - \eta(14 - 13\eta)})$.

(c) We compare q_R^{ES} and q_R^{EN}, and we can show that $q_R^{\mathrm{ES}} - q_R^{\mathrm{EN}}$ is equal to a positive factor multiplied by $g_3 = k(3k-4) + \eta + 5k\eta - 2\eta^2$. Given k_{min}, we obtain $g_3 < 0$. We compare $q_M^{\mathrm{ES}*}$ and $q_M^{\mathrm{EN}*}$, and we obtain the following: $q_M^{\mathrm{ES}*} - q_M^{\mathrm{EN}*} = \frac{k(3k+5\eta-4)\xi\sqrt{1-\sigma^2}\sigma_a}{2(k(3k-4)+6k\eta-\eta^2)} > 0$.

(d) We compare $\Pi_P^{\mathrm{ES}*}$ and $\Pi_P^{\mathrm{EN}*}$, and we obtain the following: $\Pi_P^{\mathrm{ES}*} - \Pi_P^{\mathrm{EN}*} = \frac{k(3k+6\eta-4)(1-\sigma^2)\sigma_a^2}{4(k(3k-4)+6k\eta-\eta^2)} > 0$. We compare $\Pi_M^{\mathrm{ES}*}$ and $\Pi_M^{\mathrm{EN}*}$, and we can show that $\Pi_M^{\mathrm{ES}*} - \Pi_M^{\mathrm{EN}*}$ is equal to $g_4 = \eta^2(k+\eta-1)^3 + (1-2\eta)(k(3k-4) + 6k\eta - \eta^2)^2$. There are two cases.

Case1: When 0< $\eta < \frac{1}{2}$, we require $g_4 > 0$.

Case2: When $\frac{1}{2} < \eta < 1$, given k_{min}, we require $g_4 < 0$. This completes the proof. □

B.3. Proof of Proposition 3

(a) We compare $e^{\mathrm{EN}*}$ and $e^{\mathrm{NN}*}$, and we can show that $e^{\mathrm{EN}*} - e^{\mathrm{NN}*}$ is equal to a positive factor multiplied by $g_5 = 2(k-\eta)(k(3k-4) + 6k\eta - \eta^2)\xi\sqrt{1-\sigma^2}\sigma_a - (k+\eta-1)(3k^2 - 2k(2-\eta) + (2-\eta)\eta)a_0$.

Given a_0, we can look at g_5 as a function of a_0 and $\frac{\partial g_5}{\partial a_0} = -(k+\eta-1)(3k^2 - 2k(2-\eta) + (2-\eta)\eta)$. We let $g_5 = 0$, and we obtain the solution as $\frac{2(k-\eta)(k(3k-4)+6k\eta-\eta^2)\xi\sqrt{1-\sigma^2}\sigma_a}{(k+\eta-1)(3k^2-2k(2-\eta)+(2-\eta)\eta)}$. We let $g_6 = -(3k^2 - 2k(2-\eta) + (2-\eta)\eta)$, and we can look at g_6 as a quadratic function of k and g_6 is concave.We let $g_6 = 0$, and we obtain the two solutions as $\frac{1}{3}\left(2 - \eta - \sqrt{4 - 10\eta + 4\eta^2}\right)$ and $\frac{1}{3}\left(2 - \eta + \sqrt{4 - 10\eta + 4\eta^2}\right)$. There are two cases.

Case1: When 0< $\eta < \frac{1}{2}$, there are two subcases: (i) When $k_{min} < k < k_2$, we require $g_6 > 0, a_1 < 0$, then we require $g_5 > 0$. (ii) When $k > k_2$, we require $g_6 < 0$, then we require $g_5 > 0$ if $a_0 < a_1$ and $g_5 < 0$ if $a_0 > a_1$.

Case2: When $\frac{1}{2} < \eta < 1$, there are two subcases: (i) When $k_{min} < k < \eta$, we require $g_5 < 0$. (ii) When $k > \eta$, we require $g_5 > 0$ if $a_0 < a_1$ and $g_5 < 0$ if $a_0 > a_1$ .Where $k_2 = \frac{1}{3}\left(2 - \eta + \sqrt{4 - 10\eta + 4\eta^2}\right)$, $a_1 = \frac{2(k-\eta)(k(3k-4)+6k\eta-\eta^2)\xi\sqrt{1-\sigma^2}\sigma_a}{(k+\eta-1)(3k^2-2k(2-\eta)+(2-\eta)\eta)}$.

(b) We compare $w^{\mathrm{EN}*}$ and $w^{\mathrm{NN}*}$, and we obtain the following: $w^{\mathrm{EN}*} - w^{\mathrm{NN}*} = -\frac{\eta(k+\eta-1)a_0}{k(3k-4)+6k\eta-\eta^2} < 0$. This completes the proof. □

B.4. Proof of Proposition 4

(a)We compare $e^{\mathrm{ES}*}$ and $e^{\mathrm{NS}*}$, and we can show that $e^{\mathrm{ES}*} - e^{\mathrm{NS}*}$ is equal to a positive factor multiplied by g_6. There are two cases.

Case1: When 0< $\eta < \frac{1}{2}$, we require $g_6 > 0$ if $k_{min} < k < k_2$ and $g_6 < 0$ if $k > k_2$.

Case2: When $\frac{1}{2} < \eta < 1$, we require $g_6 < 0$.

(b) We compare $w^{\mathrm{ES}*}$ and $w^{\mathrm{NS}*}$, and we obtain the following: $w^{\mathrm{ES}*} - w^{\mathrm{NS}*} = -\frac{\eta(k+\eta-1)\left(a_0+\xi\sqrt{1-\sigma^2}\sigma_a\right)}{k(3k-4)+6k\eta-\eta^2} < 0$. This completes the proof. □

B.5. Proof of Proposition 5

We compare $\Pi_M^{\mathrm{EN}*}$ and $\Pi_M^{\mathrm{NN}*}$, $\Pi_M^{\mathrm{ES}*}$ and $\Pi_M^{\mathrm{NS}*}$, and we can show that $\Pi_M^{\mathrm{EN}*} - \Pi_M^{\mathrm{NN}*}$ and $\Pi_M^{\mathrm{ES}*} - \Pi_M^{\mathrm{NS}*}$are equal to a positive factor multiplied by $g_7 = 4 + 3k^2 - (6-\eta)\eta - k(7-6\eta)$. Given η , we can look at g_7 as a quadratic function of k and g_7 is convex. We let $g_7 = 0$, and we obtain the two solutions as $\frac{1}{6}(7 - 6\eta - \sqrt{1 - 12\eta(1-2\eta)})$ and $\frac{1}{6}(7 - 6\eta + \sqrt{1 - 12\eta(1-2\eta)})$. There are three cases.

Case1: When 0< $\eta < \frac{1}{12}(3+\sqrt{3})$, the two solutions are all samller than k_{min}, thus we require $g_7 > 0$.

Case2: When $\frac{1}{12}(3+\sqrt{3}) < \eta < \frac{1}{2}$, the two solutions are all greater than k_{min}, thus there are two subcases: (i)When $k_{min} < k < k_3$ or $k > k_4$, we require $g_7 > 0$.(ii) When $k_3 < k < k_4$, we require $g_7 < 0$.

Case3: When $\frac{1}{2} < \eta < 1$, only the second solution is greater than k_{min}, thus there are two subcases: (i)When $k_{min} < k < k_4$, we require $g_7 < 0$.(ii) When $k > k_4$, we require $g_7 > 0$. Where $k_3 = \frac{1}{6}(7 - 6\eta - \sqrt{1 - 12\eta(1 - 2\eta)})$, $k_4 = \frac{1}{6}(7 - 6\eta + \sqrt{1 - 12\eta(1 - 2\eta)})$. This completes the proof. □

B.6. Proof of Proposition 6

(a)We compare $\Pi_P^{\mathrm{EN}*}$ and $\Pi_P^{\mathrm{NN}*}$, and we can show that $\Pi_P^{\mathrm{EN}*} - \Pi_P^{\mathrm{NN}*}$ is equal to $g_{10} = Aa_0^2 + C$, where $A = 2(k+\eta-1)^2(4(2k-1)\eta^2(k+\eta-1) + (k(3k-4) + 6k\eta - \eta^2)^2)$, $C = 4(3k^2 - k(4-6\eta) - \eta^2)^2(2 + k^2 - (4-\eta)\eta - k(4-6\eta))(1-\sigma^2)\sigma_a^2$, Given k , we can look at g_{10} as a quadratic function of a_0 and g_{10} is convex. We let $g_{11} = 2 + k^2 - (4-\eta)\eta - k(4-6\eta)$, and we can look at g_{11} as a quadratic function of k and g_{11} is convex. We let $g_{11} = 0$, and we obtain the two solutions as $2 - 3\eta - \sqrt{2}(1-2\eta)$ and $2 - 3\eta + \sqrt{2}(1-2\eta)$. There are two cases.

Case1: When 0< $\eta < \frac{1}{2}$, the second solution is greater than k_{min}, thus there are two subcases: (i)When $k_{min} < k < k_5$, we require $g_{11} < 0$, then there exists a_2 satisfying $a_2 > 0$ and $g_{10} = 0$ such that $g_{10} < 0$ if $a_0 < a_2$ and $g_{10} > 0$ if $a_0 > a_2$. (ii) When $k > k_5$, we require $g_{10} > 0$.

Case2: When $\frac{1}{2} < \eta < 1$, we require $g_{10} > 0$. Where $k_5 = 2 - 3\eta + \sqrt{2}(1-2\eta)$.

(b) We compare $\Pi_P^{\mathrm{ES}*}$ and $\Pi_P^{\mathrm{NS}*}$, and we can show that $\Pi_P^{\mathrm{ES}*} - \Pi_P^{\mathrm{NS}*}$ is equal to a positive factor multiplied by $g_{12} = 4(2k-1)\eta^2(k+\eta-1) - (k(3k-4) + 6k\eta - \eta^2)^2$. Given k_{min} and η, we can look at g_{12} as a function of k, which has the following properties (i) $\frac{\partial g_{12}{}^4}{\partial k^4} < 0$.(ii) $\frac{\partial g_{12}{}^3}{\partial k^3} < 0$. (iii) $\frac{\partial g_{12}{}^2}{\partial k^2} < 0$. (iv) $\frac{\partial g_{12}}{\partial k} < 0$. (v)When 0< $\eta < \frac{1}{2}$, $\lim_{k \to k_{min}} g_{12} > 0$. Then there exists k_6 such that $g_{12} > 0$ if $k_{min} < k < k_6$ and $g_{12} < 0$ if $k > k_6$.When $\frac{1}{2} < \eta < 1$, $\lim_{k \to k_{min}} g_{12} < 0$. Then we require $g_{12} < 0$. This completes the proof. □

B.7. Proof of Proposition 7

(a)We compare $w^{\mathrm{AS}*}$ and $w^{\mathrm{AN}*}$, and we obtain the following: $w^{\mathrm{AS}*} - w^{\mathrm{AN}*} = \frac{1}{2(k(3-\lambda)-1)(1+k-\lambda)(2k-\lambda)}\left(k(1-\lambda)\left(k(6k - 2(2+k)\lambda + \lambda^2) - 2(1-\lambda)\right)\xi\sqrt{1-\sigma^2}\sigma_a\right) > 0$.

(b) We compare $e^{\mathrm{AS}*}$ and $e^{\mathrm{AN}*}$, and we can show that $e^{\mathrm{AS}*} - e^{\mathrm{AN}*}$ is equal to $g_{13} = 2(1-\lambda) - k(2 + k(3-\lambda)(2-\lambda) - 2(3-\lambda)\lambda)$. We can look at g_{13} as a quadratic function of k and g_{13} is concave. We let $g_{13} = 0$, and we obtain the two solutions as $-\frac{1-(3-\lambda)\lambda+\sqrt{13-(4-\lambda)\lambda(7-(4-\lambda)\lambda)}}{(3-\lambda)(2-\lambda)}$ and $-\frac{1+(3-\lambda)\lambda-\sqrt{13-(4-\lambda)\lambda(7-(4-\lambda)\lambda)}}{(3-\lambda)(2-\lambda)}$. There are two cases.

Case1: When 0< $\lambda < \frac{1}{2}(5-\sqrt{17})$, the two solutions are all smaller than $\bar{k}$, thus we require $g_{13} < 0$.

Case2: When $\frac{1}{2}(5-\sqrt{17}) < \lambda < 1$, the second solution is greater than $\bar{k}$, thus there are two subcases: (i)When $\bar{k} < k < k_7$, we require $g_{13} > 0$.(ii) When $k > k_7$, we require $g_{13} < 0$.Where $k_7 = -\frac{1+(3-\lambda)\lambda-\sqrt{13-(4-\lambda)\lambda(7-(4-\lambda)\lambda)}}{(3-\lambda)(2-\lambda)}$. This completes the proof. □
