# Supplementary material for: Information sharing and channel structure in e-commerce supply chain considering data-driven marketing
Source: PLoS One. 2025 Sep 8;20(9):e0328040. doi: 10.1371/journal.pone.0328040 (PMC12416664; doi:10.1371/journal.pone.0328040)
Supplement: Attachment — Submitted filename: Response to Reviewers.docx [file pone.0328040.s002.docx]

**Journal Requirements:**

1. Please ensure that your manuscript meets PLOS ONE's style requirements, including those for file naming. The PLOS ONE style templates can be found at https://journals.plos.org/plosone/s/file?id=wjVg/PLOSOne_formatting_sample_main_body.pdf and [https://journals.plos.org/plosone/s/file?id=ba62/PLOSOne_formatting_sample_title_authors_affiliations.pdf](https://track.editorialmanager.com/CL0/https:%2F%2Fjournals.plos.org%2Fplosone%2Fs%2Ffile%3Fid=ba62%2FPLOSOne_formatting_sample_title_authors_affiliations.pdf/1/010f0196e6aeae99-efd8249f-9345-48e5-a04f-93b2afd710ad-000000/axXeMBhF6nRTiz4sTxqNWf8LjhyZRF-GedE5UfS4J-Q=211)

**The author’s answer:** The manuscript has been revised according to the PLOS ONE style templates as requested.

2. Please note that PLOS ONE has specific guidelines on code sharing for submissions in which author-generated code underpins the findings in the manuscript. In these cases, we expect all author-generated code to be made available without restrictions upon publication of the work. Please review our guidelines at https://journals.plos.org/plosone/s/materials-and-software-sharing#loc-sharing-code and ensure that your code is shared in a way that follows best practice and facilitates reproducibility and reuse.

**The author’s answer:** There is no author-generated code included in the manuscript.

3. Thank you for stating the following financial disclosure:
“Shanghai University of Finance and Economics Graduate Innovation Fund (Grant No. CXJJ-2021-379)”

Please state what role the funders took in the study. If the funders had no role, please state: "The funders had no role in study design, data collection and analysis, decision to publish, or preparation of the manuscript."
If this statement is not correct you must amend it as needed.
Please include this amended Role of Funder statement in your cover letter; we will change the online submission form on your behalf.

**The author’s answer:** The funders had no role in study design, data collection and analysis, decision to publish, or preparation of the manuscript.

4. Thank you for stating the following in the Acknowledgments Section of your manuscript:
“This research was funded by Shanghai University of Finance and Economics Graduate Innovation Fund (Grant No. CXJJ-2021-379).”

We note that you have provided funding information that is not currently declared in your Funding Statement. However, funding information should not appear in the Acknowledgments section or other areas of your manuscript. We will only publish funding information present in the Funding Statement section of the online submission form.
Please remove any funding-related text from the manuscript and let us know how you would like to update your Funding Statement. Currently, your Funding Statement reads as follows:
“Shanghai University of Finance and Economics Graduate Innovation Fund (Grant No. CXJJ-2021-379)”

Please include your amended statements within your cover letter; we will change the online submission form on your behalf.

**The author’s answer:** We have removed all funding-related text from the manuscript.

The Funding Statement now reads as follows:

“Zhejiang Provincial Regular Subjects of Philosophy and Social Science Planning (25NDJC104YB)”

5. We note that your Data Availability Statement is currently as follows: All relevant data are within the manuscript and its Supporting Information files

Please confirm at this time whether or not your submission contains all raw data required to replicate the results of your study. Authors must share the “minimal data set” for their submission. PLOS defines the minimal data set to consist of the data required to replicate all study findings reported in the article, as well as related metadata and methods (https://journals.plos.org/plosone/s/data-availability#loc-minimal-data-set-definition).
For example, authors should submit the following data:
- The values behind the means, standard deviations and other measures reported;
- The values used to build graphs;
- The points extracted from images for analysis.
Authors do not need to submit their entire data set if only a portion of the data was used in the reported study.
If your submission does not contain these data, please either upload them as Supporting Information files or deposit them to a stable, public repository and provide us with the relevant URLs, DOIs, or accession numbers. For a list of recommended repositories, please see https://journals.plos.org/plosone/s/recommended-repositories.
If there are ethical or legal restrictions on sharing a de-identified data set, please explain them in detail (e.g., data contain potentially sensitive information, data are owned by a third-party organization, etc.) and who has imposed them (e.g., an ethics committee). Please also provide contact information for a data access committee, ethics committee, or other institutional body to which data requests may be sent. If data are owned by a third party, please indicate how others may request data access.

**The author’s answer:** No new data were generated or analyzed in this study; therefore, data sharing is not applicable to this article.

6. When completing the data availability statement of the submission form, you indicated that you will make your data available on acceptance. We strongly recommend all authors decide on a data sharing plan before acceptance, as the process can be lengthy and hold up publication timelines. Please note that, though access restrictions are acceptable now, your entire data will need to be made freely accessible if your manuscript is accepted for publication. This policy applies to all data except where public deposition would breach compliance with the protocol approved by your research ethics board. If you are unable to adhere to our open data policy, please kindly revise your statement to explain your reasoning and we will seek the editor's input on an exemption. Please be assured that, once you have provided your new statement, the assessment of your exemption will not hold up the peer review process.

**The author’s answer:** No new data were created or analyzed in this study. Data sharing is not applicable to this article.

7. Please ensure that you refer to Figure 6 in your text as, if accepted, production will need this reference to link the reader to the figure.

**The author’s answer:** We have added a description of Figure 6 to the manuscript.

8. Please review your reference list to ensure that it is complete and correct. If you have cited papers that have been retracted, please include the rationale for doing so in the manuscript text, or remove these references and replace them with relevant current references. Any changes to the reference list should be mentioned in the rebuttal letter that accompanies your revised manuscript. If you need to cite a retracted article, indicate the article’s retracted status in the References list and also include a citation and full reference for the retraction notice.

**The author’s answer:** We have reviewed the reference list to ensure that it is complete and accurate..

**Additional Editor Comments:**

Reviewer #1:
The authors develop a game-theoretic model to investigate the encroachment and information sharing decisions considering data-driven marketing develop a game-theoretic model to investigate the encroachment and information sharing decisions considering data-driven marketing. They mathematically derive the results in the paper, and present the derivations in the appendix.
Reviewer 2:
This is a well-written and well-structured manuscript that presents a novel and significant contribution to the field. The methodology is sound, and the results are clearly presented. I recommend it for publication with minor revisions.I suggest the authors address the following points to further strengthen the manuscript:
1.Increase the proportion of recent references — The literature review would benefit from the inclusion of more up-to-date references, particularly from the past two years. This will help situate the current work more clearly within the latest developments in the field.
2.Clarify and highlight the marginal contribution — While the core idea is interesting, the marginal contribution over existing work could be more explicitly emphasized. The authors are encouraged to clearly articulate what distinguishes their approach from closely related studies, both in the introduction and conclusion sections.
Once these points are addressed, I would be happy to recommend the manuscript for publication.
Reviewer 3:
The manuscript is well-organized and clearly written. It provides a thorough and insightful analysis of channel choice and information-sharing decisions in the e-commerce supply chain. The methodology is appropriate, and the results are sound and well-supported. The work represents a valuable contribution to the field.

**The author’s answer:** We have updated the data in the background section to reflect the most recent available year and increased the proportion of recent references. Additionally, we have added a description of the marginal contribution in the Introduction section.
